# ChatGPT and Occupational Therapy: A Study of Generated Program Feasibility

**DOI:** 10.7759/cureus.83761

**Published:** 2025-05-08

**Authors:** Tadatoshi Inoue, Shogo Sawamura, Tatsuya Sera, Takahiro Takenaka, Kengo Kohiyama, Takashi Nagai

**Affiliations:** 1 Department of Rehabilitation, Heisei College of Health Sciences, Gifu, JPN

**Keywords:** chatgpt-4 evaluation, creating a plan, large language models, occupational therapy program, program generation

## Abstract

Introduction

As the application of large language models (LLMs) in the medical field advances, the potential for creating occupational therapy (OT) programs remains unexplored. This study aimed to clarify the ability of Generative Pre-trained Transformer (GPT; OpenAI, San Francisco, CA, USA) to create OT programs.

Methods

Based on five case reports of patients with stroke and concomitant psychological symptoms, GPT was instructed to create OT programs. Five occupational therapists (OTRs) evaluated the generated programs and quantified the degree of agreement with the programs created by OTRs.

Results

The programs generated by GPT showed a low degree of agreement with the programs created by OTRs in all cases, with a rating of two points or less. The content was found to be general, lacking in specificity and individuality, and insufficiently specialized. The scores for all programs generated by GPT were 2/4 or lower.

Discussion

While GPT has difficulty creating OT programs based on patients' life backgrounds and specialized knowledge, it showed the potential to be used in some processes of OT program creation. This is likely due to a lack of pretraining and limitations in information.

## Introduction

In recent years, there have been numerous reports on the successful application of large language models (LLMs) in the medical field [1,2]. Examples of LLMs include ChatGPT-4 (OpenAI, San Francisco, CA, USA), Google's Bard (Google, Mountain View, CA, USA), and Microsoft's Bing Chat (Microsoft Corp., Redmond, WA, USA), which are rapidly gaining popularity due to their advanced natural language processing capabilities. In particular, Generative Pre-trained Transformer (GPT) developed by OpenAI is freely accessible and capable of performing a wide range of tasks beyond text generation [3]. Moreover, it has demonstrated usefulness in solving physical therapy licensing examination questions, reducing interpretation variability, and improving efficiency in medical image diagnosis [4,5]. Its usefulness and convenience have led to efforts to apply GPT in creating rehabilitation programs. For example, Zhang et al. [6] used GPT to formulate a general rehabilitation plan and generate International Classification of Functioning, Disability and Health (ICF) codes for a textbook-based stroke case. Similarly, Mittal and Dhar [7] reported that GPT reduced the time required to create rehabilitation programs for six elderly individuals; however, there were limitations in terms of accuracy, indicating the need for final confirmation by physicians and therapists. Occupational therapy (OT), in particular, presents unique challenges for automation. The work of occupational therapists (OTRs) is considered difficult to replicate with artificial intelligence (AI) due to the complexity of their tasks [8]. OT emphasizes the importance of understanding an individual's life background and developmental history to tailor interventions that support their specific way of living. This high degree of individuality contributes to the complexity of OT program development. While several studies have explored the use of GPT in general medical and rehabilitation contexts, to date, no research has specifically examined its ability to generate occupational therapy programs. Therefore, the purpose of this study was to evaluate GPT’s capability in creating OT intervention plans.

## Materials and methods

Case reports of occupational therapy

The case reports analyzed were selected from the Japanese Journal of Occupational Therapy, the official journal of the Japanese Society of Occupational Therapy, based on the following criteria: the primary author is an OTR; the primary disease of the case is stroke with comorbid depressive or motivational symptoms; the intervention was conducted in an inpatient rehabilitation ward; and the PDF is available online. They must be available as PDFs for online viewing and published in or after 2021. Five articles that met these criteria were selected for analysis [9-13], which are summarized below.

Case Report 1

A male patient in his 50s developed mild motor paralysis and apraxia due to a cerebral hemorrhage and subsequently experienced a decline in mood. The OT program utilized motor imagery and compensatory strategies for antagonistic apraxia and intermittent difficulty initiating movement in daily life situations. Additionally, psychological care was provided to improve self-esteem [9].

Case Report 2

A female patient in her 70s developed moderate motor paralysis, aphasia, sensory impairment, and depression due to a cerebral infarction. The OT program focused on upper limb functional training using a MOMO orthosis (Reharo Co., Tokyo, Japan) and strengthening self-efficacy, aiming for the gradual reacquisition of household activities and social roles. Furthermore, emotional improvement was addressed through successful training experiences. MOMO is an arm support that utilizes a bracket to be fixed to a desk, with the forearm resting on an elbow rest from above. It supports the upper limb at a constant height and can assist with horizontal and upward reaches [10].

Case Report 3

In this case report, the gender and age were not specified. The patient developed mild motor paralysis and depression due to a cerebral infarction. The OT program aimed to improve basic movements such as standing and walking, activities of daily living such as dressing and toileting, cognitive function through puzzles and coloring, and mental function through karaoke and small group activities [11].

Case Report 4

A female patient in her 70s developed moderate motor paralysis, sensory impairment, and depression due to a cerebral hemorrhage. The OT program aimed to recover from paralysis through electrical stimulation therapy. Additionally, it aimed to improve motivation for OT using visual feedback. Activities of daily living training using assistive devices was conducted to promote independence in daily life [12].

Case Report 5

A female patient in her 80s developed severe motor paralysis and depression due to a cerebral infarction. The OT program aimed to recover from paralysis through electrical stimulation therapy. Training in activities of daily living, such as grooming and toileting, as well as laundry folding and shopping training, was conducted to improve independence in daily life activities. Furthermore, psychological support was provided by closely listening to and empathizing with the patient's mental state [13].

Input to GPT

After removing personal information from the case introduction and initial evaluation in each article, it was inputted into GPT. The following prompt was additionally inputted: "Please formulate an occupational therapy (OT) program for the following patient. Please present the OT program in a table format, specifying the training name, training content, duration, frequency, and load." In this study, a newly created GPT account was used, and the task was performed using a zero-shot prompt (a method of generating output directly without prior learning or adjustment) without any prior learning about OT. GPT version 4 was used, and the input was in Japanese.

Analysis

The intervention content presented in each article and the OT program created by GPT were organized in the format of training name, content, duration, frequency, and load. To compare these, the evaluation was conducted by five OTRs with more than 10 years of experience. The areas of expertise for each OTR are as follows: OTR(A) specializes in the mental health field, OTR(B) in the physical disabilities field, OTR(C) in the physical disabilities field, OTR(D) in the higher brain dysfunction field, and OTR(E) in the geriatric disabilities field. The evaluation method was as follows: 4 points for complete agreement, 3 points for substantial agreement, 2 points for partial agreement, 1 point for substantial disagreement, and 0 points for complete disagreement. Comments on specific comparison points were also collected and analyzed. Furthermore, we collected comments specifically on the points of comparison and analyzed those as well. Regarding the translation process, the manuscript was initially drafted in Japanese by the authors. Subsequently, the authors meticulously translated the manuscript into English. During this process, great care was taken to ensure clarity and appropriate English expression for the academic readership in this field while maintaining the original meaning and context of the Japanese text.

## Results

Table 1 shows the training name, content, duration, frequency, and load generated by GPT after inputting the evaluation details of each case report. GPT was able to faithfully create the program according to the prompts.

**Table 1:** Occupational therapy programs created by ChatGPT Enter the evaluation results in the case report into ChatGPT, and indicate the name of the training, training content, duration, frequency, and load.

	Training name	Training content	Duration	Frequency	Load
Case report 1 [9]	Approach for counteracting apraxia (left-hand reflex control training)	Intentional control and awareness training to reduce counteractive movements of the left hand; practice suppressing the left hand during right-hand movements under the therapist's support	4 weeks	5 times/week	30 minutes/day; gradually reducing support for inhibition
	Daily living activity training (eating, grooming, hygiene, dressing training)	Gradually restrict the use of the left hand during daily activities (e.g., eating, dressing, and grooming); use assistive devices as needed in the initial stages to reduce finger grasping	8 weeks	5 times/week	15 minutes per activity; extend the time based on progress
	Training to suppress compulsive grasp reflex (reflex control training with sensory stimulation)	Training to suppress the grasp reflex using sensory stimulation (e.g., cooling and vibration); muscle relaxation training to reduce muscle tension	6 weeks	3 times/week	20 minutes per session; adjust load based on response
	Approach for intermittent movement initiation difficulty (movement initiation promotion training)	Training with sequential movement instructions and visual feedback to facilitate movement initiation; break down movements and practice sequentially in order	10 weeks	3 times/week	30 minutes per session; gradually add steps until movements are autonomous
	Emotional and cognitive approach (depression symptom alleviation training)	Provide psychological support and motivation, focusing on positive behaviors; introduce relaxation techniques for cognitive-behavioral therapy and stress reduction	12 weeks	2 times/week	60 minutes per session; dialogue and practice with the therapist
Case report 2 [10]	Upper limb and finger function training	Joint range of motion (ROM) training for the upper limb	6 weeks	5 times/week	10-15 minutes/day
		Strength training (lifting fingers and upper limb, resistance exercises)	8 weeks	5 times/week	5-10 reps/set, two sets
		Functional movement training (using occupational activities)	8 weeks	3 times/week	20-30 minutes/day
	Sensory function training	Tactile recognition training (tactile awareness training)	6 weeks	3 times/week	10-15 minutes/day
		Perception training (discrimination of object weight and shape)	6 weeks	3 times/week	10-15 minutes/day
		Multisensory integration training (combining visual and tactile senses)	8 weeks	2 times/week	20 minutes/day
	Higher brain function training	Attention training (enhancing focus using visual and auditory stimuli)	12 weeks	3 times/week	20-30 minutes/day
		Memory training (recalling simple daily tasks)	12 weeks	3 times/week	20-30 minutes/day
		Language training (supporting basic communication)	12 weeks	3 times/week	20-30 minutes/day
	Daily living activity training	Basic daily activities (mobility, eating, dressing)	12 weeks	5 times/week	1-2 times/day (for each activity)
		Instrumental activities of daily living (IADL) training (household tasks like cooking and cleaning)	12 weeks	3 times/week	20-30 minutes/session
		Hobby activity resumption support (e.g., handicrafts)	12 weeks	2 times/week	30 minutes/session
	Behavioral and emotional support	Environmental adjustment (ensuring safety and reducing stress)	Ongoing	As needed	Continuous
		Emotional support (addressing anxiety and restless behaviors)	Ongoing	As needed	Continuous
Case report 3 [11]	Apathy and post-stroke depression (PSD) mitigation training	Introduction of cognitive-behavioral therapy; reintroduction of hobby activities to stimulate cognitive function	12 weeks	3 times/week	60 minutes/session; gradually increasing conversation topics and activities
	Spontaneity improvement training	Environmental adjustments and training to enhance spontaneity in daily activities; gradual self-care training	8 weeks	5 times/week	30 minutes/session; progression evaluated and adjusted per self-care task
	Walking and mobility independence training	Continued walking training using a walker; balance training to prevent falls	10 weeks	3 times/week	30 minutes/session; gradually increasing walking distance (up to over 100 meters)
	Caregiver support program	Care education to reduce caregiver burden; building a cooperative system with family, sharing strategies for reducing BPSD through dialogue	6 weeks	Once/week	60 minutes/session; including family counseling and suggestions for specific caregiving methods
	Conversation increase training	Improving communication skills through regular conversations and activities with relatives; approaching past topics using reminiscence therapy	12 weeks	Once/week	45 minutes/session; conversation topics adjusted according to individual adaptation
Case report 4 [12]	Voluntary movement promotion training	Promoting voluntary movement of the paretic upper limb through mirror therapy and repetitive facilitation exercises; joint mobilization and muscle strengthening exercises	12 weeks	5 times/week	30 minutes per session; difficulty is gradually increased (light load in early stages, higher load in later stages)
	Support training for activities of daily living (ADL)	Reacquisition of eating, dressing, and grooming skills; use and training of assistive devices	10 weeks	4 times/week	45 minutes per session; progress is based on the patient's ability with appropriate levels of support
	Apathy and post-stroke depression (PSD) reduction training	Cognitive-behavioral therapy (CBT) and motivational interviewing; introduction of hobby activities to rekindle interest	12 weeks	2 times/week	45 minutes per session; gradually increasing activities to enhance motivation
	Mental stability and relaxation	Anxiety reduction through deep breathing exercises and progressive muscle relaxation techniques; introduction of physical and mental relaxation	8 weeks	3 times/week	20 minutes per session; session duration is increased based on progress
	Community activity reintegration training	Gradual support for reintegration into community activities; promotion of interaction with family and community members	16 weeks	Once/week	60 minutes per session; gradually increasing the frequency and distance of outings
Case report 5 [13]	Upper limb function training	Range of motion training for the left upper limb and shoulder girdle	6 weeks	5 days/week	30 minutes per session; repetitive passive and assisted movements, aiming to improve shoulder girdle stability
		Sensory stimulation training for the left hand and fingers	4 weeks	5 days/week	20 minutes per session; using tactile and vibration stimuli to increase sensory input
	Standing and walking training	Walking training using a walker	8 weeks	5 days/week	30 minutes per session; improving left-right balance and propulsion with a walker
		Step exercise	6 weeks	3 days/week	15 minutes per session; seated stepping exercises to enhance lower limb strength and circulation
	Activities of daily living (ADL) training	Training for independence in eating	4 weeks	5 days/week	20 minutes per session; practicing spoon and fork usage with simple assistive devices
		Training for independence in dressing	6 weeks	5 days/week	30 minutes per session; focusing on approaches to the paralyzed side and utilizing the unaffected side
	Cognitive training	Attention function enhancement training	8 weeks	3 days/week	30 minutes per session; conducting cognitive tasks based on the trail making test (TMT)
		Frontal lobe function enhancement training	8 weeks	3 days/week	30 minutes per session; providing tasks related to the frontal assessment battery (FAB)
	Social participation promotion	Household simulation training	8 weeks	1 day/week	40 minutes per session; simulating household tasks and gradually regaining roles
		Support for resuming community activities	12 weeks	1 day/week	40 minutes per session; assisting with resumption of regional activities and family interactions

**Figure 1:**
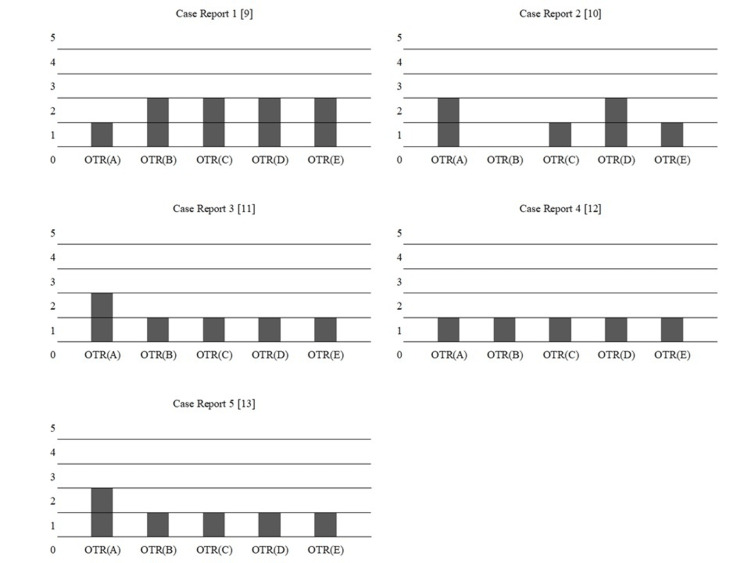
OTR scoring of programs created by ChatGPT Each case report includes evaluations from five occupational therapists (OTRs). The vertical axis represents the score, and the horizontal axis represents each occupational therapist.

Table 2 shows the quantitative evaluation results of the OT programs from the papers and the OT programs generated by GPT. Figure 1 shows a graph of the evaluations for each case. For all case reports, the evaluation by each OTR was 2 points or less, indicating substantial to complete disagreement. This clearly shows a discrepancy between the OT programs created by OTRs and those generated by GPT.

**Table 2:** Occupational therapy program score sheet created by ChatGPT The results were rated as follows: 4 = completely consistent, 3 = generally consistent, 2 = partially consistent, 1 = generally inconsistent, 0 = completely inconsistent.

	OTR(A)	OTR(B)	OTR(C)	OTR(D)	OTR(E)
Case report 1 [9]	1	2	2	2	2
Case report 2 [10]	2	0	1	2	1
Case report 3 [11]	2	1	1	1	1
Case report 4 [12]	1	1	1	1	1
Case report 5 [13]	2	1	1	1	1

Table 3 summarizes the comments made when comparing the OT programs from the papers and the OT programs generated by GPT. The programs generated by GPT lacked specificity and individuality and tended to be general. Additionally, it was shown that they did not include novel or highly specialized interventions.

**Table 3:** Comments on the occupational therapy program created by ChatGPT Five occupational therapists shared their opinions comparing the training content for case reports they developed with training content generated by ChatGPT. IVE: immersive virtual environment, CAOD: classification and other data, ADOC: aid for decision-making in occupation choice.

Case report	OTR(A)	OTR(B)	OTR(C)	OTR(D)	OTR(E)
Case report 1 [9]	The thesis customizes the approach according to the subject’s cases and individuality, and the training content is specific. GPT has broader and shallower training content compared to the thesis.	GPT does not consider the background of staff involvement and practical implementation, leading to low realism despite many variations.	GPT lacks interventions involving imagery and diverse approaches for ADL stages, assistive devices, or self-defense mechanisms.	GPT specifies intervention periods, evidence on cognitive behavioral therapy, etc., but it is not well-established.	The intervention period for the disease stage is not specified, and there is insufficient detail regarding vocational assistance and training content.
Case report 2 [10]	Some training names are consistent, but the specific content of the training differs.	There is no mention of MOMO in GPT, and MOMO outcomes are overwhelmingly inconsistent.	GPT does not integrate MOMO in its interventions.	GPT's understanding of MOMO is insufficient, likely affecting treatment strategies.	Training content is overly generalized, making it unrealistic to implement most of it practically.
Case report 3 [11]	While the paper utilizes a small group setting, GPT seems better suited for one-on-one interactions.	GPT is suitable for anyone with general applicability.	GPT integrates cognitive behavioral therapy, along with discussions involving karaoke or family.	GPT's work model is undefined, with an emphasis on medical models.	GPT does not consider the subject's daily life, and family involvement is limited, reflecting poorly on real-world application.
Case report 4 [12]	Specialized treatment methods in the thesis are less broadly applicable in GPT, with many clinic methods missing.	GPT emphasizes mental health support, with fewer standard outpatient treatments and little adherence to guidelines.	GPT training content is diverse, but there is no mention of IVE or CAOD methodologies.	GPT does not address transfer packages, lacking insight into broader training systems.	The content on transfer packages, IVES, and social participation is missing, reflecting limited application in practical scenarios.
Case report 5 [13]	The thesis focuses on the patient’s life after discharge, highlighting the individuality of training.	GPT offers generic training for diverse cases, with limited individuality.	GPT’s training program can apply broadly, but lacks details on CAOD and ADOC.	GPT has a broad range of content but fails to address specialized training like ADOC.	Training content is unrelated to IVES or CAOD and fails to consider the disease stage or subject's individuality.

## Discussion

In this study, we compared the OT programs presented in published papers with those generated by GPT, based on the same case information and initial evaluation data. The comparison revealed limited agreement between the two. However, a quantitative analysis of the content indicated some overlap, suggesting that GPT may have potential utility in certain stages of OT program development. However, the quantitative evaluation by five OTRs was 2 points or less, indicating that the content often did not agree. Moreover, the differences in the training content created by GPT can be summarized into two points: (1) the patient's life background is not considered, and the content is general, lacking specificity and individuality, and (2) novel and highly specialized interventions with limited knowledge are not created. In other words, GPT is considered vulnerable to individual differences in patients, as it is based on general information. Ohse stated that GPT has information uncertainty and that its answers tend to be general and immature in terms of professional opinions [14]. In this study, the content inputted into GPT was only textual information, and the OT programs presented in the papers were created by OTRs who deal with patients daily in the ward. Furthermore, GPT was not pretrained on stroke or impaired mental function. Therefore, it cannot consider detailed information such as the patient's personality, developmental history, and living environment that is not shown in the papers. For these reasons, it is considered that GPT is based on limited information and that the lack of practical experience has affected the inconsistency of the content. Additionally, the characteristics of GPT can be cited as the reason why it cannot create novel or highly specialized OT programs with limited knowledge. As of October 2023, GPT relies on information from publicly available websites, books, and papers, and its vulnerability to the latest information and its lack of reliability and accuracy have been reported [15,16]. Therefore, while the content of the training items was consistent, we believe a discrepancy arose between the papers and the programs created by GPT in terms of individuality. Therefore, it is considered that there is a discrepancy between the programs created by the papers and GPT. Previous studies examining treatment programs utilizing LLMs have reported that ChatGPT, in a study where it created treatment programs for oncology cases, lacked individualization and faced difficulty in obtaining patient consent [17]. Furthermore, research in the psychiatric domain that used GPT to generate treatment plans indicated a lack of accuracy and utility [18], suggesting that it cannot replace medical professionals, struggles with medical judgment, and should be used cautiously in clinical applications [19]. In this study, the OT program generated by GPT for stroke patients with comorbid psychological symptoms lacked individualization and deviated from the program actually implemented by the OTR. Therefore, we believe that programs created by GPT cannot be directly applied to patients and require the support and supervision of an OTR. Rather than using GPT as a substitute tool for creating OT programs, we consider it important to leverage it as a supportive tool to aid thinking, such as summarizing literature, confirming practical examples of OT programs, and generating multiple OT programs from case information for reference.

Limitations and future directions

This study has four limitations and future directions. (1) The LLM used in this study was the free version of GPT. Therefore, the OT program was created with limited GPT functionality. In the future, it is necessary to create an OT program using the paid version and conduct follow-up research. On the other hand, this study demonstrated its usefulness for low- and middle-income groups. Additionally, we have not created OT programs using other LLMs such as OpenAI o1 or Bard. In the future, it is important to compare these LLMs to determine which LLM is most similar to the programs created by OTRs. (2) The number of papers used for the analysis was small. Therefore, reproducibility and validity are not ensured. It is important to re-examine this by increasing the number of model case reports in the future. (3) GPT was not pretrained on OT or rehabilitation for patients with stroke and impaired mental function. It is speculated that the created program may change by conducting few-shot learning on GPT or carefully examining the prompt content. In the future, it is important to pretrain GPT with case presentations and carefully examine the prompt content before inputting it. (4) Since the prompt inputted into GPT is not confidential, ethical issues cannot be ruled out. While it is acceptable to use simulated case studies for research and educational purposes, it is difficult to use actual case information as it is. Therefore, further discussion is needed to develop guidelines for clinical application.

## Conclusions

This study investigated the utility of GPT in creating OT programs. The results revealed that there was little agreement when compared to the OT programs created by OTRs based on case reports. The characteristics of the programs generated by GPT included a lack of individuality and specialization, and the content was generally generic. The results suggest that the clinical application of GPT remains challenging with the current version and the prompt content used. On the other hand, there is still a possibility that accuracy can be improved by training GPT with model cases and corresponding OT programs and then inputting detailed prompts. In the future, it is important to examine the learning effects of GPT.
